# Enhanced Magnetic Properties of FeSiAl Soft Magnetic Composites Prepared by Utilizing PSA as Resin Insulating Layer

**DOI:** 10.3390/polym13091350

**Published:** 2021-04-21

**Authors:** Hao Lu, Yaqiang Dong, Xincai Liu, Zhonghao Liu, Yue Wu, Haijie Zhang, Aina He, Jiawei Li, Xinmin Wang

**Affiliations:** 1 Faculty of Materials Science and Chemical Engineering, Ningbo University, Ningbo 315211, China; luhao@nimte.ac.cn (H.L.); liuzhonghao@nimte.ac.cn (Z.L.); 2 Zhejiang Province Key Laboratory of Magnetic Materials and Application Technology, CAS Key Laboratory of Magnetic Materials and Devices, Ningbo Institute of Materials Technology & Engineering, Chinese Academy of Sciences, Ningbo 315201, China; wuyue@nimte.ac.cn (Y.W.); zhanghaijie@nimte.ac.cn (H.Z.); heaina@nimte.ac.cn (A.H.); lijiawei@nimte.ac.cn (J.L.); wangxinmin@nimte.ac.cn (X.W.); 3 University of Chinese Academy of Sciences, Beijing 100049, China

**Keywords:** soft magnetic composites, poly-silicon-containing arylacetylene resin, stability, core loss

## Abstract

Thermosetting organic resins are widely applied as insulating coatings for soft magnetic powder cores (SMPCs) because of their high electrical resistivity. However, their poor thermal stability and thermal decomposition lead to a decrease in electrical resistivity, thus limiting the annealing temperature of SMPCs. The large amount of internal stress generated by soft magnetic composites during pressing must be mitigated at high temperatures; therefore, it is especially important to find organic resins with excellent thermal stabilities. In this study, we prepared SMPCs using poly-silicon-containing arylacetylene resin, an organic resin resistant to high temperatures, as an insulating layer. With 2 wt % PSA as an insulating layer and annealed at 700 °C for 1 h, the FeSiAl SMPCs achieved the best magnetic properties, including the lowest core loss of 184 mW/cm^3^ (measured at 0.1 T and 50 kHz) and highest permeability of 96.

## 1. Introduction

Metal soft magnetic powder cores (SMPCs) are prepared by traditional powder metallurgy by mixing a metal magnetic powder and an insulating medium. Because of the addition of an insulating medium, SMPCs exhibit high resistivity and low loss [1,2,3,4,5]. The power density and switching frequency of SMPCs are between those of silicon steel and ferrite, which just fills the gap during this period. Therefore, they are widely used in main transformers, high-frequency chokes, automobiles, large-capacity switching power supplies, and electromagnetic chargers. The increasing transmission efficiency in the 5G era requires magnetic materials that can operate at higher frequencies. However, the eddy-current loss (*P*_cv_) of soft magnetic materials at high frequencies cannot be avoided. *P*_cv_ generates a large amount of heat and causes the magnetic performance to decrease, which greatly restricts applications in high-end products. The key to reducing *P*_cv_ is to prepare an insulating layer with a high resistivity and strong binding force to the magnetic powder [6,7]. Many attempts have been made to select insulating coating materials, which are mainly categorized as organic or inorganic. Polyimide, phenol, epoxy, parylene, silicone, and acrylic resins have been used as organic coating materials for SMPCs and amorphous ribbons [8,9,10,11,12,13,14,15,16,17,18,19]. The advantage of organic coatings is their good coating integrity, but their heat-resistant temperatures are limited, generally less than 500 °C. Amorphous ribbons require polymers with multiple functional properties such as high insulation, corrosion resistance, increased sensitivity, and high-temperature resistance. Thus, existing polymers require further research and development. Inorganic coating materials include phosphate, oxides, sodium silicate, and ferrite [20,21,22,23,24,25,26,27,28,29].

To fully release the stress generated during the pressing process, SMPCs generally require heat treatment at temperatures above 600 °C. High-temperature heat treatment destroys the structure of the organic coating layer and increases *P*_cv_ between the magnetic powder particles, which is particularly severe at high frequencies. To improve the soft magnetic properties, we must develop new insulating coating materials with high resistivity, thermal stability, and bonding strength.

Poly-silicon-containing arylacetylene resin (PSA), a kind of organic–inorganic hybrid polymer, has the advantages of high electrical insulation, super high thermal stability, and excellent bonding strength [30,31], making it an ideal magnetic powder core insulating material. However, there are no related reports on the preparation of SMPCs using PSA as an insulating layer. In this study, to further improve the high thermal stability and magnetic properties of FeSiAl SMPCs, PSA was used as an insulating layer, and the effects of the PSA content and heat treatment on the magnetic properties of the SMPCs were systematically examined.

## 2. Materials and Experimental Method

Gas-atomized FeSiAl (85 wt % Fe–9.6 wt % Si–5.4 wt % Al) powder with an average particle size of 45 µm was purchased from Changsha Hualiu Powder Metallurgy Company. PSA was provided by the Special Functional Polymer Laboratory of the East China University of Science and Technology. Epoxy (EP) and silicone resin (SI) were supplied by Shandong Haoshun Chemical. A silane coupling agent (KH550), which was used as a surface pretreatment for the magnetic particles to enhance their binding strength with the organic resin, was supplied by Nanjing Shuguang Chemical Group.

The FeSiAl powders were surface-treated in KH550 at 1% of the magnetic particle mass. Subsequently, PSA was dissolved in acetone at mass ratios of 1, 2, 3, and 4 wt % relative to the FeSiAl powder and mechanically stirred for 0.5 h. The mixture was then stirred under ultrasonic conditions for 2 h, and the solution temperature was maintained below 60 °C. The hybrid magnetic particles were then dried in a vacuum oven at 120 °C for 24 h. For comparison, we prepared magnetic powders coated with SI and EP resin with a mass ratio of 2 wt % relative to the FeSiAl powder. The magnetic particles were pressed and shaped at 1800 MPa to obtain annular samples (20.3 mm outer diameter, 12.7 mm inner diameter, and 5.0 mm height). To study the effect of the annealing temperature, the ring-shaped samples were annealed at 400, 500, 600, and 700 °C for 1 h. In addition, the performances of the three resin-coated powder cores were compared at 600 °C.

The surface morphology of the powdered core samples after annealing was observed using scanning electron microscopy (SEM, EVO18). The relative molecular mass and distribution of the PSA were measured using a gel permeation chromatograph (HLC 8320, Tosoh, Japan) with high-performance liquid-chromatography-grade tetrahydrofuran as the solvent. The thermal resistance of the PSA as an insulating layer was studied using thermogravimetry (TG). The characteristic functional groups of the PSA and its existence on the surface of the magnetic powders were analyzed using an intelligent Fourier infrared spectrometer (FTIR) in the range of 500 to 3500 cm^−1^. The corrosion resistances of the samples were compared using the salt-spray test, and the corrosion resistance of the heat-treated samples was tested using a salt-spray test box. The NaCl concentration in the salt-spray box was set to 0.35 wt %, and the temperature was room temperature. The corrosion potentials of the samples were tested using an electrochemical workstation. The effective permeability of the SMPCs was tested using an impedance analyzer (Agilent 4294A, America), and the power loss of the SMPCs was measured using an AC B-H loop tracer (MATS–2010SA, America) with a measurement range of 10–100 kHz. The hysteresis loops were detected using a vibrating sample magnetometer (Lakeshore 7410, America).

## 3. Results

### 3.1. Analysis of the PSA

**Figure 1:**
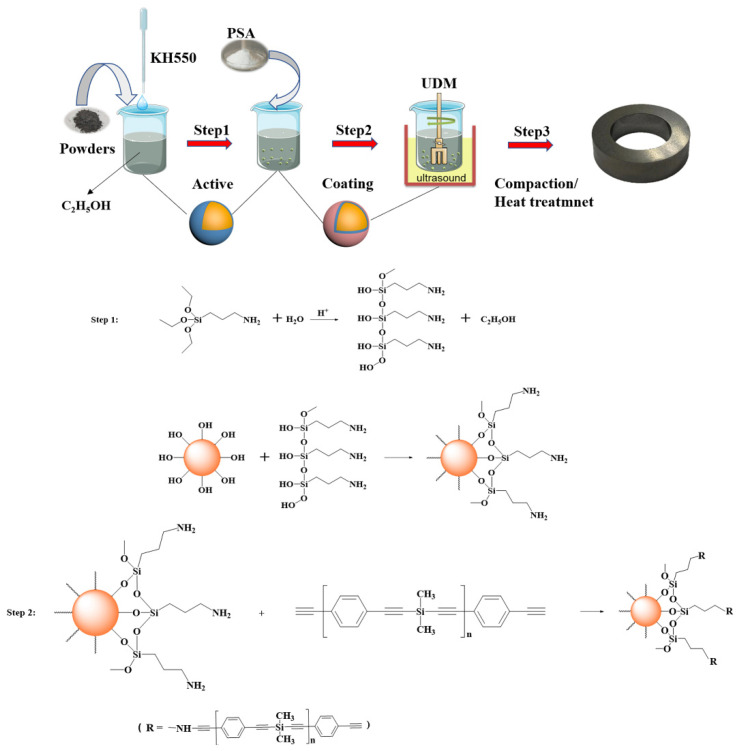
Schematic diagram of sample preparation and coating mechanism.

The sample preparation process is illustrated in Figure 1, where the coating mechanism is marked as steps 1 and 2. The silanol produced by the hydrolysis of KH550 connected to the surface of the magnetic powder through the hydroxyl group [32,33], and the end of KH550 connected to the PSA surface via intermolecular forces, thereby strengthening the binding force between the coating layer and magnetic powder [34].

**Figure 2:**
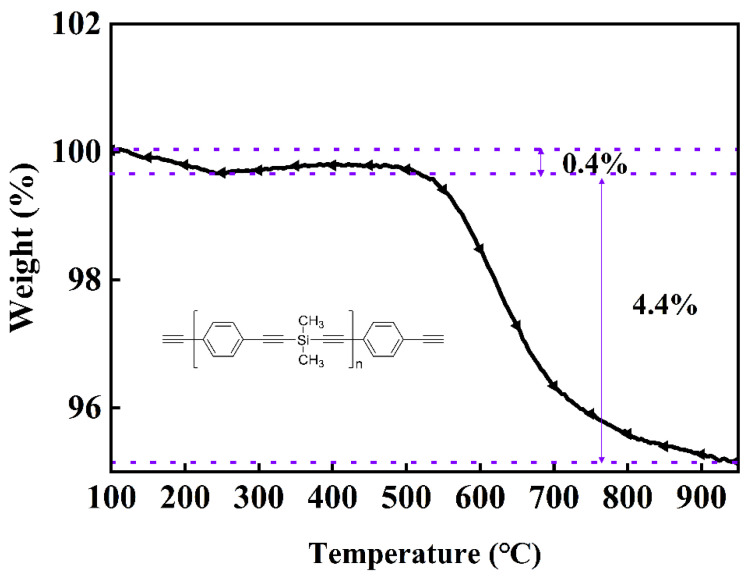
PSA structure and thermogravimetric curve.

Figure 2 shows the TG analyses of the PSA and the basic structure of the PSA measured at a heating rate of 10 °C/min in a nitrogen atmosphere. Two weight-loss stages of the PSA were detected during the entire process. The volatilization of solvent impurities at 100–300 °C resulted in a mass loss of 0.4%, and a mass loss of 4.4% in the second stage at 500–900 °C was mainly caused by the decomposition of the PSA. Considering that the heat-treatment temperature of magnetic powder cores is generally between 600 and 700 °C, this mass loss was considered completely acceptable. The results of the TG analysis showed that the PSA had excellent thermal stability. The alkynyl group of the PSA main chain underwent a crosslinking reaction under heating to form an aromatic ring structure. Rupture of the molecular chain of the crosslinked polymer required the breaking of multiple chemical bonds, which effectively inhibited the pyrolysis of PSA, and the introduction of silicon increased the thermal-decomposition temperature of the resin.

**Figure 3:**
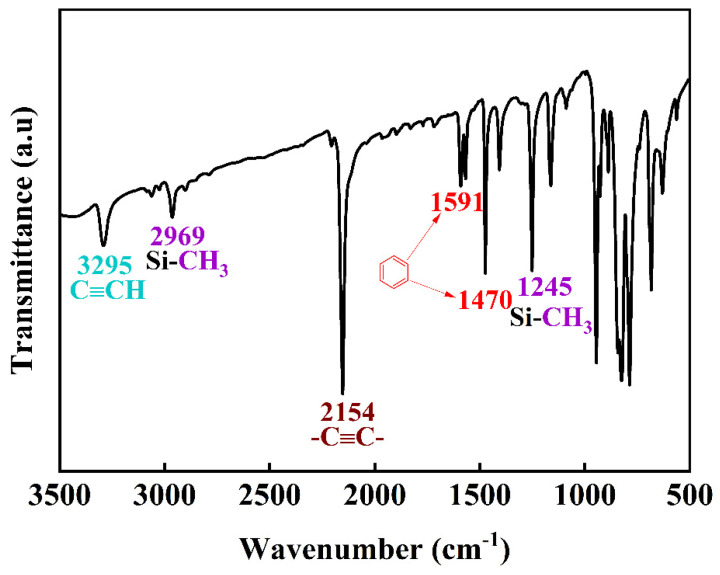
FTIR spectra for PSA.

Figure 3 shows the infrared spectrum of the PSA in the range of 500 to 3500 cm^−1^. Among the peaks, 3295 cm^−1^ is the stretching vibration of alkyne hydrogen (C≡CH), 2154 cm^−1^ is the stretching vibration of -C≡C-, 2969 cm^−1^ is the asymmetric stretching vibration of -CH_3_ in Si-CH_3_, and 1591, 1570, 1470, and 1402 cm^−1^ are the C=C skeletal stretching vibrations on the benzene ring. The peak at 1245 cm^−1^ corresponds to flexural vibration outside the -CH_3_ plane in Si-CH_3_ [30]. The relative molecular mass and distribution of PSA were measured by gel permeation chromatography. The measured M_n_ of PSA was 1804, and M_w_ was 2509. Combined with infrared analysis, these results proved that the substance was PSA.

### 3.2. Magnetic Properties

**Figure 4:**
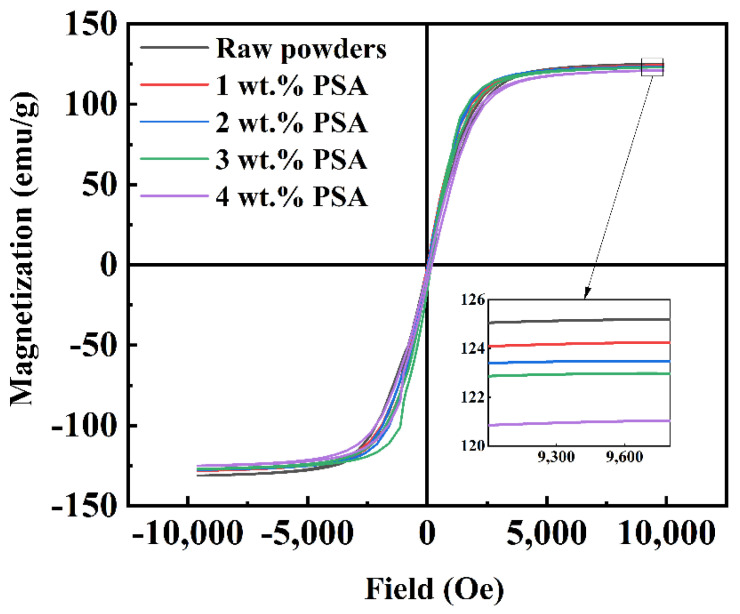
Magnetization curves of magnetic powder coat with different content of PSA.

Figure 4 shows the magnetization curves of the FeSiAl powders coated with different amounts of PSA. The saturation magnetization (*M*_s_) of the coated magnetic powder was slightly reduced, but the effect was within a controllable range and had little influence on the properties. This shows that PSA as an insulating coating material did not deteriorate *M*_s_ of the magnetic powder cores.

**Figure 5:**
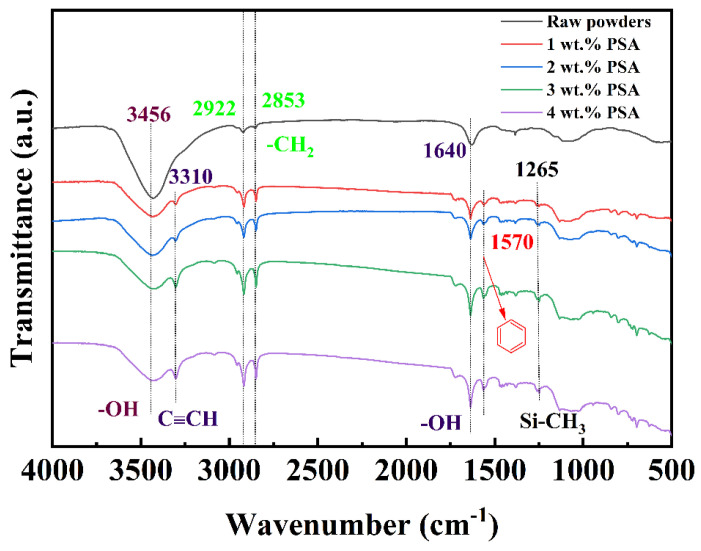
FTIR spectra for powder coated with varied content of PSA.

Figure 5 shows the FITR spectra of the magnetic powders coated with different PSA contents and the original powder. Each curve had strong absorption peaks at 3456 cm^−1^ and 1640 cm^−1^ corresponding to -OH groups, mainly due to water absorbed on the surface of the magnetic powders and the hydrolysis of KH550. The peaks at 2853 and 2922 cm^−1^ were derived from the stretching vibration of -CH_2_ bonds, and the peak at 1384 cm^−1^ was attributed to the deformation vibration of C-H. Absorption peaks belonging to PSA were detected at 3310, 1570, and 1265 cm^−1^, while these peaks were absent from the spectrum of the original powder. It can be concluded that PSA was coated onto the surface of the magnetic powders.

**Figure 6:**
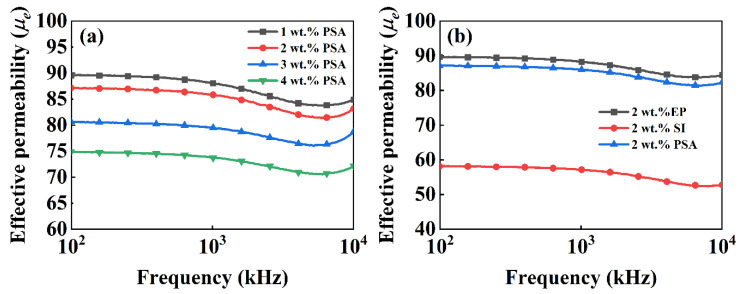
The effective permeability of different content (**a**) and different resins (**b**).

**Figure 7:**
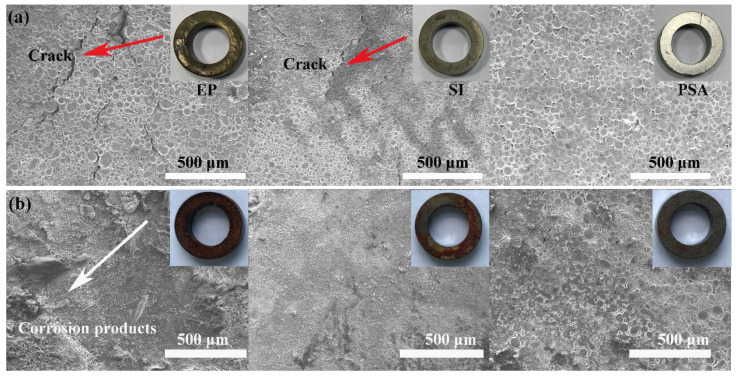
Surface morphology of the sample after annealing (**a**), and morphology after salt-spray corrosion (**b**).

Figure 6a,b show the effective permeability (*μ_e_*) of the samples coated with 1–4 wt % PSA contents and 2 wt % EP and SI after heat treatment at 600 °C. At a resin content of 2 wt %, the PSA-coated sample had a medium *μ_e_* of 87 at 1 MHz. In contrast, the SI-coated sample had the lowest *μ_e_* of 53 at 1 MHz. The EP-coated samples showed the highest magnetic permeability, largely because EP has poor thermal stability, and significant decomposition causes the volume fraction of the nonmagnetic phase to decrease, resulting in a higher magnetic permeability. This indicates that the PSA had a weak magnetic dilution effect and was suitable for use as an insulating coating material. With increasing PSA content, *μ_e_* of the samples at 1 MHz decreased from 89 to 74. However, the frequency stability improved at the same time. There are two main reasons for this. The magnetic permeability of magnetic powder cores can be calculated by the following equation [35]:

(1) $$\mu_{e}=1+\frac{4\pi \left(\mu -1\right)}{4\pi +N\left(\mu -1\right)}\left(1-p\right),$$

where *μ* is the intrinsic permeability of the material, *N* is the demagnetization factor, and *p* is the percentage of nonmagnetic materials in the composite. Based on Equation (1), the magnetic permeability of the magnetic powder cores continuously decreased with increasing nonmagnetic materials. On the other hand, with increasing PSA content, the particle surface thickness became uneven. As a result, the combination of powders was looser, the gaps between particles increased, and the density decreased, thereby reducing the effective magnetic permeability of the powder cores. Figure 7a shows the appearance of the samples after heat treatment at 600 °C. The EP-coated sample had serious cracks, the SI-coated sample had slight cracks, and the PSA-coated sample had a smooth surface. This is because EP and SI have poor thermal stabilities; they decompose rapidly at high temperatures, and the generated gas escapes and destroys the original topographic structure.

To intuitively compare the corrosion resistances of the samples, a 24 h salt-spray test was performed, and the results are shown in Figure 7b. The EP-coated samples were severely corroded, with a thick antirust layer that easily fell off; the SI-coated samples were moderately corroded; and the PSA-coated samples were only slightly corroded. This is directly related to the thermal stability of the resins. The insulating layer of the EP-coated sample was incomplete and thin after heat treatment, resulting in poor oxygen concentrations in different areas on the surface of the magnetic powder. Therefore, Cl^−^ easily adsorbed onto the surface defects and ultimately caused crevice corrosion. The PSA decomposed only slightly during heat treatment, and thus it had excellent corrosion resistance.

**Figure 8:**
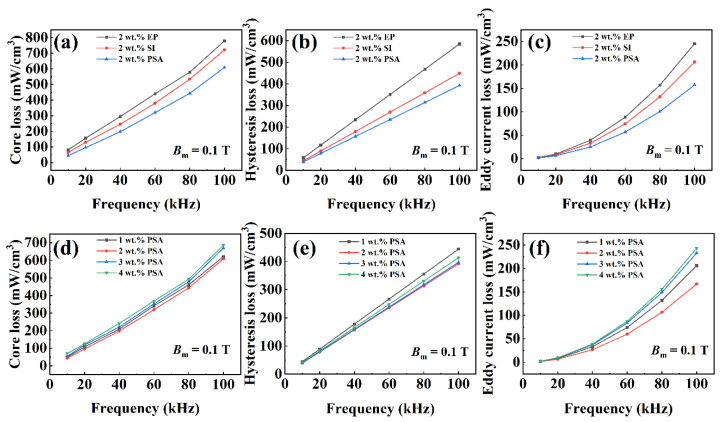
The total core loss (**a**,**d**), hysteresis loss (**b**,**e**), and eddy-current loss (**c**,**f**) as function of the frequency for the samples.

Figure 8a,d show the core loss (*P*_cv_) of the samples coated with 1–4 wt % PSA and 2 wt % EP and SI after heat treatment at 600 °C. The 2 wt % PSA sample had the lowest *P*_cv_ of 319 and 608 mW/cm^3^ at 60 and 100 kHz, respectively. To explore how the loss was reduced, we performed a loss-separation analysis, and the hysteresis loss (*P*_h_) and eddy-current loss (*P*_e_) after separation are shown in Figure 8b,c and Figure 8e,f, respectively. Under a magnetic field of 0.1 T, *P*_h_ of the samples was not significantly different, although that of the PSA-coated samples was slightly lower. *P*_h_ was dominant in the low-frequency range and mainly originated from the rotation of the magnetic domain during the magnetization process. The magnitude of *P*_h_ was proportional to the area of the DC hysteresis loop, which can be expressed by the following empirical formula:

(2) $$P_{h}=K_{h}B_{m}^{\alpha }f,$$

where *K*_h_ is the hysteresis coefficient, *B_m_* is the maximum induction field or test field strength, ƒ is the test frequency, and *α* is the simulation coefficient. Both *K*_h_ and *α* are related to the coercivity of the material itself, and the coercivity of the powder cores can be reduced by annealing heat treatment and a uniform insulating coating. Under the same heat-treatment temperature, *P*_h_ will not make much difference. *P_e_* dominates at higher frequencies and is proportional to the square of ƒ. It is generally composed of interparticle eddy-current loss (*P_inter_*) and intraparticle eddy-current loss (*P_intra_*), and can be expressed as [25,36,37]:

(3) $$P_{e}=P_{inter}+P_{intra}=\left(\frac{h^{2}}{\beta \rho_{SMCs}}+\frac{d}{20\rho_{powder}}\right)\pi^{2}B_{m}^{2}f^{2},$$

where *h* is the thickness of the sample, *d* is the diameter of the magnetic powder particles, *β* is the geometric coefficient, and *ρ_SMCs_* and *ρ_powder_* are the resistivities of the powder core and the magnetic powder, respectively. Among them, the particle-size ratio used in this study is beneficial for reducing *P_inter_.* Compared with the samples coated with PSA, the samples coated with EP and SI resins had higher *P*_e_ values of 88 and 74 mW/cm^3^, respectively, because of their poor thermal stability. The resins decomposed at high temperatures and destroyed the coating layer. The above results indicate that a coating with an appropriate PSA content is beneficial in obtaining SMPCs with low-loss characteristics.

**Figure 9:**
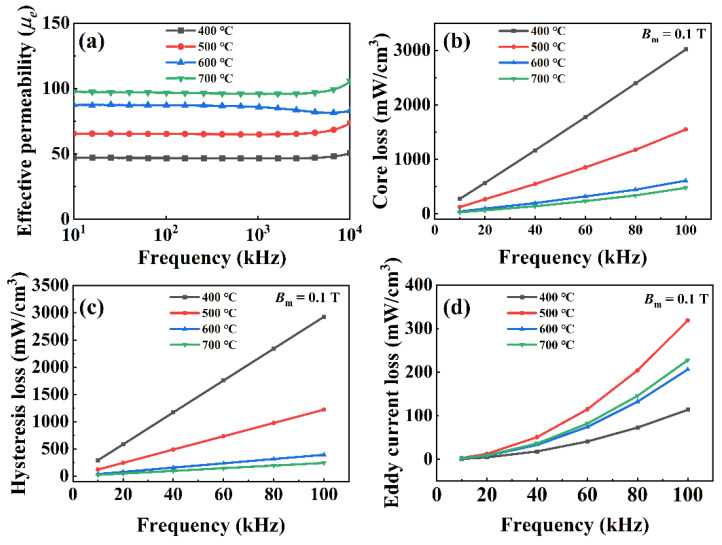
The effective permeability (**a**), the total core loss (**b**), hysteresis loss (**c**), eddy-current loss (**d**) as a function of the frequency for the samples.

In summary, at a heat-treatment temperature of 600 °C, the 2 wt % PSA-coated sample had the lowest *P*_cv_. Therefore, we used 2 wt % PSA-coated samples to explore the effect of the heat-treatment temperature on the magnetic properties. The effective permeability, core loss, and loss separation of the samples annealed at 400–700 °C are shown in Figure 9. The magnetic permeability continued to rise for two reasons. First, high-temperature heat treatment eliminated the internal stress generated during the pressing process, removed the pinning effect of internal stress on the domain walls, reduced the rotation resistance of the magnetic domain, and reduced *P*_h_. The higher the temperature was, the more internal stress was released, and the better the magnetic properties of the powder core. However, there was also a limit to how much the magnetic properties could be improved by high temperatures. If the temperature was too high, overburning occurred, destroying the insulating layer and increasing *P*_e_. PSA has a higher temperature limit because of its excellent heat resistance. The other reason for the increasing magnetic permeability was that the slight PSA decomposition reduced the volume fraction of the nonmagnetic phase. The core loss of the sample at 10–100 kHz continued to decrease, mainly due to the decrease in *P*_h_. *P*_e_ did not decrease with increasing temperature, which may have been caused by the increase in *P*_e_ between particles caused by the partial decomposition of the PSA. At 700 °C, the powder core had the best magnetic performance, with an effective permeability of 96 at 1 MHz and core loss of 184 mW/cm^3^ (measured at 50 kHz and 0.1 T).

## 4. Conclusions

The PSA showed excellent thermal stability and had little effect on *M*_s_ of the magnetic powders. Thus, it can be used as an insulating coating material for SMPCs. Owing to the high thermal stability of the PSA, it remained on the surface of the magnetic powder after heat treatment, which not only increased the allowable heat-treatment temperature and permeability of the powder core, but also reduced the core loss. The samples coated with 2 wt % PSA and treated at 700 °C achieved the best magnetic properties, with a maximum permeability of 96 and *P*_cv_ of 184 mW/cm^3^ (measured at 50 kHz and 0.1 T). In addition, the corrosion resistance of the powder core was significantly improved.

## Data Availability

The data presented in this study are available on request from the corresponding author.
